# Multi-Locus Genome-Wide Association Studies Reveal Fruit Quality Hotspots in Peach Genome

**DOI:** 10.3389/fpls.2021.644799

**Published:** 2021-02-25

**Authors:** Cassia da Silva Linge, Lichun Cai, Wanfang Fu, John Clark, Margaret Worthington, Zena Rawandoozi, David H. Byrne, Ksenija Gasic

**Affiliations:** ^1^Department of Plant and Environmental Sciences, Clemson University, Clemson, SC, United States; ^2^Department of Horticulture, Michigan State University, East Lansing, MI, United States; ^3^Department of Horticulture, University of Arkansas, Fayetteville, AR, United States; ^4^Department of Horticultural Sciences, Texas A&M University, College Station, TX, United States

**Keywords:** FarmCPU, mrMLM 4.0, candidate gene analyses, SNP array, RosBREED, QTN

## Abstract

Peach is one of the most important fruit crops in the world, with the global annual production about 24.6 million tons. The United States is the fourth-largest producer after China, Spain, and Italy. Peach consumption has decreased over the last decade, most likely due to inconsistent quality of the fruit on the market. Thus, marker-assisted selection for fruit quality traits is highly desired in fresh market peach breeding programs and one of the major goals of the RosBREED project. The ability to use DNA information to select for desirable traits would enable peach breeders to efficiently plan crosses and select seedlings with desired quality traits early in the selection process before fruiting. Therefore, we assembled a multi-locus genome wide association study (GWAS) of 620 individuals from three public fresh market peach breeding programs (Arkansas, Texas, and South Carolina). The material was genotyped using 9K SNP array and the traits were phenotyped for three phenological (bloom date, ripening date, and days after bloom) and 11 fruit quality-related traits (blush, fruit diameter, fruit weight, adherence, fruit firmness, redness around pit, fruit texture, pit weight, soluble solid concentration, titratable acidity, and pH) over three seasons (2010, 2011, and 2012). Multi-locus association analyses, carried out using mrMLM 4.0 and FarmCPU R packages, revealed a total of 967 and 180 quantitative trait nucleotides (QTNs), respectively. Among the 88 consistently reliable QTNs detected using multiple multi-locus GWAS methods and/or at least two seasons, 44 were detected for the first time. Fruit quality hotspots were identified on chromosomes 1, 3, 4, 5, 6, and 8. Out of 566 candidate genes detected in the genomic regions harboring the QTN clusters, 435 were functionally annotated. Gene enrichment analyses revealed 68 different gene ontology (GO) terms associated with fruit quality traits. Data reported here advance our understanding of genetic mechanisms underlying important fruit quality traits and further support the development of DNA tools for breeding.

## Introduction

Peach [*Prunus persica* (L.) Batsch] is a diploid species, with a short juvenile period (2–4 years), relatively simple genome (∼230 Mbp), and one of the best genetically characterized deciduous trees (Verde et al., 2013). Peach is the third most cultivated temperate tree fruit in the world, after apple and pear, with a world production of approximately 24.6 million tons (Food and Agricultural Organization of the United Nations (FAOSTAT), 2018). Despite the high production, peach consumption has declined over the past decades. In the United States, peach per capita consumption decreased to 1.3 kg per year compared to ∼3 kg per year in the 1980s (Minas et al., 2018). Inconsistent and low fruit quality is recognized as the major limiting factor for consumer acceptance and, consequently, the low rates of peach consumption (Cirilli et al., 2016).

Peach breeders have always selected for fruit quality with respect to size, color and firmness, as well as tried to expand harvest season (Laurens et al., 2018). Recently, more emphasis is on other traits such as internal quality and postharvest traits (Elsadr et al., 2019).

Recent advances in next-generation high-throughput sequencing and genotyping techniques, such as development of the 9K peach SNP array by the International Peach SNP Consortium (IPSC) (Verde et al., 2012), allow use of DNA information to develop tools for facilitating breeding efforts (Lambert et al., 2016; da Silva Linge et al., 2018). Understanding the genetic mechanisms that control a specific trait would enable peach breeders to efficiently apply marker-assisted breeding (MAB) through the development of DNA diagnostic tools, and consequently select seedlings with desired quality traits early in the selection process before the characters can be evaluated in the field (Abdelghafar et al., 2020).

The link between the genetic markers and a particular trait could be determined using different approaches. Quantitative trait loci analysis (QTL mapping) and genome-wide association studies (GWAS) are widely used for dissection of complex genetic traits (Meneses and Orellana, 2013). In peach, several linkage maps have been used in QTL discovery of key fruit quality traits such as fruit size, diameter, firmness, acidity, soluble solid concentration, individual sugars, maturity date, pubescence, blush, fruit texture, and phytochemical compounds (Eduardo et al., 2011; Martínez-García et al., 2013; Pirona et al., 2013; Frett et al., 2014; Vendramin et al., 2014; da Silva Linge et al., 2015; Zeballos et al., 2016; Ciacciulli et al., 2018; Nuñez-Lillo et al., 2019; Abdelghafar et al., 2020). These maps were typically developed for mapping particular traits in a specific parental background with limited recombination events and genetic diversity.

Alternatively, GWAS has the advantage of increasing the recombination events and consequently mapping resolution with a significant reduction of the research time (Zhu et al., 2008). However, false positives due to population structure or kinship among genotypes, or false negatives due to removal of rare alleles that are involved in natural variation are some of the weaknesses of GWAS (Brachi et al., 2011). To deal with this problem, GWAS methods utilizing mixed linear models (MLM), which take into account multiple levels of relatedness, have become standard methodology (Yu et al., 2006). Significant marker-trait association based on the single-locus models, such as the general linear model (GLM) and MLM, were reported for several traits such as fruit pubescence, fruit shape, stone adhesion-flesh texture, fruit flesh color, non-melting/melting flesh, fruit weight, titratable acidity, soluble solid concentration, leaf gland type, flower type, bloom date, fruit development period, maturity date, ripening index, and total sugars (Micheletti et al., 2015; Cao et al., 2016, 2019; Elsadr et al., 2019; Font I Forcada et al., 2019). Single-locus models test one locus at a time and fail to match the true genetic model of complex traits that are controlled by numerous loci simultaneously (Xu et al., 2018). Thus, major improvements in GWAS statistical methodology have occurred, and multi-locus GWAS methods considering the information of all loci simultaneously have been developed (Wang et al., 2016).

Recently, six multi-locus GWAS approaches were integrated into an R package, named mrMLM (Zhang et al., 2020). The mrMLM 4.0 R package comprises the mrMLM (Wang et al., 2016), FASTmrMLM (Tamba and Zhang, 2018), FASTmrEMMA (Wen et al., 2017), ISIS EM-BLASSO (Tamba et al., 2017), pLARmEB (Zhang et al., 2017), and pKWmEB (Ren et al., 2018) two-step multi-locus GWAS methods. First, various algorithms are used to select all potentially associated markers. Second, these selected markers are put in one model, in which all the effects are obtained by empirical Bayes, and all the non-zero effects are further identified by likelihood ratio test for true Quantitative Trait Nucleotides (QTNs) (Zhang et al., 2020).

The multi-locus model Fixed and random model Circulating Probability Unification (FarmCPU) uses the associated markers as covariates in a fixed-effect model (FEM) and optimization on the associated covariate markers in a random effect model (REM). FarmCPU adopts REML optimization to replace the criterion that the variance explained by kinship is near zero, which can only be arbitrarily determined. FarmCPU also adopted a binning approach from super to select pseudo QTNs. The whole genome is equally divided into bins, and only one significant marker with the smallest *P*-value from each bin is selected as the candidate pseudo QTN. These candidate pseudo QTNs are determined by a REM. The candidate pseudo QTNs are first ranked by *P*-value. Then, the best combinations between the different bins and the number of candidate pseudo QTNs are determined by REM. Finally, the two types of models (FEM and REM) are performed iteratively until no change occurs in the selection of pseudo QTNs (Huang et al., 2018). Thus, FarmCPU decreases the computer time required, provides reliable results by efficiently removing the confounding between the population structure and Kinship, avoiding model over-fitting, and controlling for false positives (Liu et al., 2016).

The objective of this study was to identify significant marker-trait association for 14 agronomic traits, using the multi-locus GWAS methods in mrMLM 4.0 and FarmCPU in a U.S. peach diversity germplasm panel of 620 individuals, managed by three public fresh market peach breeding programs at University of Arkansas System Division of Agriculture, Texas A&M University and Clemson University.

## Materials and Methods

### Plant Material, DNA Isolation, Quantification, and Genotyping

The material used in this study represents the U.S. peach breeding germplasm assembled under the RosBREED project (Iezzoni et al., 2010, 2020; Peace et al., 2014). A total of 72 cultivars/advanced selections and 548 individuals from three public fresh market peach breeding programs: University of Arkansas System Division of Agriculture (AR), Clemson University (SC), and Texas A&M University (TX), were chosen to effectively represent alleles currently found within North American fresh market peach breeding germplasm (Supplementary Table 1).

Peach DNA was extracted from young leaves using the E-Z 96 Tissue DNA Kit (Omega Bio-Tek, Inc., Norcross, GA, United States). DNA was quantitated with the QuantiT PicoGreen Assay (Invitrogen, Carlsbad, CA, United States), using the Victor multi-plate reader (Perkin Elmer Inc., San Jose, CA, United States). The final DNA concentrations were adjusted to a minimum of 50 ng/μL and submitted to the Research Technology Support Facility at Michigan State University (East Lansing, MI, United States).

Samples were genotyped with the IPSC peach 9K SNP array v1 (Verde et al., 2012). The SNP data curation was performed using the workflow for high-resolution genetic marker data described in Vanderzande et al. (2019). After the SNP data curation, a total of 4005 SNPs distributed over the eight peach chromosomes remained and were used in the multi-locus GWAS (Supplementary Table 2).

### Phenotypic Data

Phenotypic data were recorded over three seasons (2010–2012) at each fresh market peach program. Bloom data (BD; Julian days) were visually assessed in the field and recorded for each tree when 60–80% of the blossoms were open. Ripening date (RD; Julian days) was determined when 20% of fruits were at commercial harvest by visually inspecting the presence of a few soft fruits in the field for maturity two times per week. Days after bloom (DAB; Julian days) was calculated as the number of days between the date of full bloom and ripening date.

Approximately 20 fruits were harvested for phenotyping. A five firm fruit sample was selected for the following traits evaluations: Blush (0–5 scale, 0 = none, and 3 = 40–60%, 5 > 90% red blush on fruit surface) subjective scales were used as described by Frett et al. (2014). Fruit diameter (FDIA; mm) was evaluated with a millimeter caliper, while fruit weight (FW; g) was measured as the average weight of the five selected peaches. Flesh adherence (ADH) was evaluated using 1 - 4 scale where 1 = Freestone; 2 = Semi-freestone; 3 = Semi-clingstone; and 4 = Clingstone. Fruit firmness (FF; N) was measured using an electronic fruit texture analyzer (FTA) fitted with an 8-mm diameter tip (GÜSS Fruit Texture Analyzer; GÜSS Manufacturing (Pty) Ltd., Strand, South Africa). All readings were recorded as kilogram-force (kgf) and then converted to Newton (N) by multiplying the reading by 9.807. Redness around Pit (RP) was measured following the scale 1 = red; 0 = no red. The fruit texture (FT) was evaluated using the scale 1 = melting; 2 = non-melting. Pit weight (PW; g) was measured as the average weight of the five selected pits.

For biochemical traits, a composite sample of one approximately 2 cm wide longitudinal slice from each of the five fruits was used to extract juice with a juicer for the measurement of soluble solid concentration (SSC) using a digital refractometer, pH with a pH meter and titratable acidity (TA) using an automatic titrator (DL 22 Food and Beverage analyzer, Mettler Toledo, Columbus, OH, United States). TA was obtained by the titration of solution of 6 g of the peach juice diluted with 50 mL of distilled water to pH 8.2 with 0.1N NaOH and expressed as milliequivalents of malic acid. The following equation was used to calculate titratable acidity (the milliequivalent factor used corresponded to malic acid, 0.067):


$$Titratableacidity\left(\%\right)=\frac{\left[N\,a\,O\,H\,t\,i\,t\,r\,a\,t\,e\,d\,\left(m\,l\right)\times 0.1\,N\,\left(N\,a\,O\,H\right)\times m\,i\,l\,l\,i\,e\,q\,u\,i\,v\,a\,l\,e\,n\,t\,f\,a\,c\,t\,o\,r\times 100\right]}{6\,g\,o\,f\,j\,u\,i\,c\,e}$$

### Descriptive Analysis, Genetic Diversity, and Population Structure

The descriptive analysis and the correlations between the traits were performed using the software Past (Hammer et al., 2001). The genetic diversity analysis was performed using the GenAlEx software (Peakall and Smouse, 2012). The narrow sense heritability was calculated using the R package Sommer (Covarrubias-Pazaran, 2016) using the h2.fun:


h⁢2.f⁢u⁢n⁢(o⁢b⁢j⁢e⁢c⁢t,d⁢a⁢t⁢a,g⁢T⁢e⁢r⁢m,e⁢T⁢e⁢r⁢m)

where: object represents a model fitted with the mmer function; data represents the dataset used to fit the model provided in the object argument; gTerm is a character vector specifying the genetic terms fitted in the model; and eTerm is a character vector specifying the environment term fitted in the model. For the level from the eTerm (environment) the heritability is calculated as:


$$1-\left(P\,E\,V/\left(m\,d*V_{g}\right)\right)$$

“PEV” is the predicted error variance for the genotype, “md” is the mean value from the diagonal of the relationship (genomic) matrix “G” and where “Vg” refers to the genotype variance. The model included in the h2.fun was:


mix<-mmer(Trait∼Year,

random=∼vs(ds(Year),Selection,Gu=K)+vs(ds(Local)),

rcov=∼vs(ds(Year),units),data=Trait)

where “K” refers to the genomic relationship matrix. Population structure, multidimensional scaling (MDS) and Bayesian clustering were performed with fastSTRUCTURE (Raj et al., 2014). The MDS was performed using TASSEL (Bradbury et al., 2007). The MDS results were plotted with the R package “scatterplot3D” (Ligges and Mächler, 2002). The fastSTRUCTURE was run with a “simple prior” option and remaining default parameters. The number of populations (K), ranging from 1 to 20, and the most probable number of populations was chosen for running the built-in script for multiple choices of K. The admixture proportions of each genotype, estimated by fastSTRUCTURE, were visualized using DISTRUCT plots (Rosenberg, 2004). Accessions were assigned to a specific subpopulation when the estimated membership coefficients (Q) were above 0.80.

Linkage disequilibrium (LD) was measured by correlation coefficients (*r*^2^) for all pairs of SNPs. The LD decay were calculated using PopLDdecay (Zhang et al., 2018a) with the following parameters: -MaxDist 3000 kb -MAF 0.05.

### Genome-Wide Association Study

To validate and increase the accuracy of the multi-locus GWAS results, we used mrMLM 4.0 (Zhang et al., 2020) and FarmCPU (Liu et al., 2016). The six multi-locus GWAS methods (mrMLM, FASTmrMLM, FASTmrEMMA, pLARmEB, pKWmEB, and ISIS EM-BLASSO) from mrMLM 4.0 R package were used. The SNP data were converted to character, as described in the user manual, the population structure was the Q matrix obtained from fastSTRUCTURE and the kinship matrix was calculated by mrMLM 4.0. All parameters in GWAS were set at default values. The significantly associated SNPs were determined by the critical threshold of LOD score ≥ 3 as described in previous studies (Tamba et al., 2017). Concerning FarmCPU, the SNP data were converted to numerical using the R package GAPIT (Lipka et al., 2012). Principle component analysis (PCA) was conducted using TASSEL 5.0, and the first three components were incorporated as covariates in the GWAS model. Bonferroni-corrected *P*-value threshold was set at *p* < 0.01.

We considered a QTN reliable when: QTNs repeatedly detected in at least four methods and/or two seasons using the mrMLM 4.0; QTN consistently detected in two seasons using FarmCPU; QTNs detected in at least three methods in mrMLM 4.0 and also identified in the FarmCPU approach. These QTNs were named as “qtn” + trait name abbreviation + scaffold + detected QTL order on chromosome.

### Candidate Genes

The candidate gene analysis was performed using two strategies. First, the candidate gene analyses were performed within the haploblock regions in which a QTN was detected with at least three methods in mrMLM 4.0 and with FarmCPU. Haploblock regions encompassing the associated SNPs were determined in PLINK 1.9 (Chang et al., 2015) using the flag “blocks” restricted to 500 kb. From the *Prunus persica* Whole Genome v2.0 Assembly & Annotation v2.1 (Verde et al., 2017) in Genome Database for Rosaceae^1^ (Jung et al., 2018), a systematic search was conducted to compile the predicted candidate genes associated with the quality traits. The candidate genes were further analyzed for GO (gene ontology) enrichment using GOseq 1.42.0 R package (Young et al., 2010). The GO terms were considered significantly enriched or depleted at FDR < 0.05. The enriched GO terms were visualized using REVIGO semantic similarities (Supek et al., 2011). Second, we compared the position of the already reported candidate genes responsible for regulating BD, RP (*Cs locus*), Blush, RD, pH, and TA (*D locus*) with the QTL hotspot regions detected in this study.

## Results

### Phenotypic Data

Six hundred twenty individuals from the three fresh market public peach breeding programs were evaluated for 14 different fruit quality traits over 3 years (2010–2012) (Table 1). The BD, DAB, FDIA, and TA traits were analyzed in two seasons (2011 and 2012), with the BD, DAB, and TA exhibiting the highest mean values in 2011 and FDIA in 2012. The RD, FW, FF, PW, SSC, and pH, as well as the categorical traits Blush, ADH, RP, and FT were evaluated in three seasons (2010–2012). The RD, FW, and pH varied from 111 to 237 Julian days, 30.2 – 351.4 g and 2.8 – 5.1 respectively, with the highest mean values observed in 2010 (197.6 Julian days, 119.4 g and 3.9, respectively). The FF fluctuated from 0.9 N to 106.4N with the highest mean value measured in 2011 (21.1 N). The traits PW and SSC ranged from 2.5 to 12.2 and 7.2 – 26.8, respectively, with the highest mean values in 2012 (6.8 g and 13.2).

**TABLE 1:** Descriptive analysis of 14 phenotypic traits observed in 620 individuals from three U.S. public fresh market peach breeding programs (Univ. of Arkansas, Texas A&M and Clemson Univ.) over three seasons (2010–2012).

Trait	Year	Min	Max	Mean	SE	SD
BD	2011/2012	37/36	80/78	65.1/59.31	1.14/0.51	16.19/10.93
RD	2010/2011/2012	158/111/125	230/237/230	197.6/189.1/171.3	1.2/1.1.02	14.9/23.5/22.2
DAB	2011/2012	67/78	196/159	120.8/111.3	1.52/0.82	19.98/17.24
Blush	2010/2011/2012	0/0/0	5/5/5	2.8/3.0/2.7	0.1/0.05/0.04	1.3/1.0.93
FDIA	2011/2012	36.3/42.0	85.2/80.6	55.2/60.8	0.42/0.37	7.47/6.66
FW	2010/2011/2012	49.0/33.4/30.2	214.7/351.4/289.8	119.4/103.1/117.1	2.9/1.9/1.9	34.9/40.7/42.2
ADH	2010/2011/2012	1/1/1	4/4/4	2.2/2.5454	0.1/0.07/0.06	1.4/1.4/1.4
FF	2010/2011/2012	1.4/2.1/0.9	54.6/67.9/106.4	16.2/21.1/17.6	0.9/0.6/0.7	10.5/12.0/14.8
RP	2010/2011/2012	0/0/0	1/1/1	0.8/0.8/0.6	0.04/0.02/0.02	0.4/0.4/0.5
FT	2010/2011/2012	1/1/1	2/2/2	1.1/1.3/1.3	0.02/0.03/0.03	0.3/0.7/0.7
PW	2010/2011/2012	2.6/2.5/2.8	10.7/12.2/11.6	6.0/5.9/6.8	0.1/0.1/0.1	1.8/1.9/1.6
SSC	2010/2011/2012	7.5/7.2/7.6	17.9/22.2/26.8	11.7/13.1/13.2	0.2/0.1/0.1	2.1/2.9/3.3
TA	2011/2012	0.2/0.01	1.7/1.8	0.7/0.6	0.01/0.01	0.3/0.3
pH	2010/2011/2012	3.4/2.9/2.8	5.0/4.8/5.1	3.9/3.8/3.8	0.02/0.02/0.03	0.3/0.3/0.4

*BD, bloom date (Julian days); RD, ripening date (Julian days); DAB, days after bloom (Julian days); blush (0–5 scale); FDIA, fruit diameter (mm); FW, fruit weight (g); ADH, adherence (1–4); FF, fruit firmness (N); RP, Redness around pit (0–1 scale); FT, fruit texture (1–2 scale); PW, pit weight (g); SSC, soluble solid concentration (°Brix); TA, titratable acidity (% malic acid); SE, mean standard error; SD, standard deviation.*

Highly significant (*P* < 0.01) correlations were observed between the traits (Supplementary Table 3). The highest correlation was observed between the FDIA_2011 and FW_2011 (0.92). As expected, a significant negative correlation was detected between the pH and TA (−0.65 and −0.64 in 2011 and 2012, respectively). The traits DAB and RD revealed a significant positive correlation in the years analyzed (0.82 and 0.87). Concerning the correlation between years, BD showed the highest correlation (0.99), followed by RD (0.92).

The narrow sense heritability (h^2^) was estimated for all 14 traits (Supplementary Table 4). High average values of h^2^ (> 0.6) were observed for TA (0.87), RD (0.83), BD (0.77), ADH (0.77), FT (0.76), DAB (0.74), FDIA (0.73), SSC (0.72), Blush (0.71), FW (0.70), PW (0.70), RP (0.69), pH (0.69), and FF (0.68).

### Genetic Variability, Population Structure, and Linkage Disequilibrium

The observed mean heterozygosity (Ho) per individual was 0.35, ranging from 0.06 in “St John” to 0.68 in “Elberta.” The mean Ho per SNP was 0.36 ranging from 0.07 in SNP_IGA_598267 (scaffold 5) to 0.97 in SNP_IGA_573558 (chromosome 5). The chromosome 6 was the least heterozygous (Ho = 0.281) while the chromosome 1 revealed the highest heterozygosis (Ho = 0.385). The mean expected heterozygosity (He) was 0.370, ranging from 0.08 (SNP_IGA_624226; scaffold 6) to 0.500. The mean average inbreeding coefficient (F = (Ho-He) /Ho) was 0.05, ranging from −0.949 to 0.595.

The population structure was analyzed with Multidimensional scaling (MDS) and fastSTRUCTURE. The MDS revealed two main groups, in which the second group could be divided in two clusters. The first group comprised the individuals from the TX breeding program, while the second group grouped the individuals from the AR and SC breeding programs (Supplementary Figure 1). Population structure analysis with fastSTRUCTURE suggested a number of K between 2 and 19. However, the population stratification for *K* = 3 showed clear differences between groups based mainly on the pedigree information of the individuals belonging to each group (Supplementary Figure 1). The first group accounted for the individuals related to ‘Tropic Beauty’, ‘TX2293_3’, ‘TX2B136’, ‘TXW1293_1’, and ‘TXW1490_1’. The second group comprised individuals linked to ‘A_663’, ‘A_760’, and ‘Bolinha’, while the third group contained individuals linked either to ‘Clayton’ and/or ‘O’Henry’.

The LD decayed with increase of physical distance between SNPs in all groups (Supplementary Figure 2). Considering the admixed individuals, the average of *r*^2^ was 0.16. The physical distance over which LD decayed to half of its maximum value was around 540 kb. Different patterns of LD decays were observed in the three different groups. Group 3 revealed the highest average of *r*^2^ (0.32) and the longest physical distances in which LD decayed to half of its maximum value (1,620 kb), while group 2 showed shortest distance (480 kb). In the group 1, the LD decayed of its maximum value of *r*^2^ in approximately ∼540 kb.

### Multi-Locus Genome-Wide Association Study

GWAS using the six multi-locus methods in the R package mrMLM 4.0 revealed a total of 967 QTNs associated with 14 traits (Supplementary Table 5). The highest number of associated SNPs was observed on chromosome 4 (99) and the lowest in chromosome 7 (23). Significant QTNs detected in at least four methods in the same season, were detected for almost all traits except TA. In addition, consistently associated QTNs identified in at least two seasons were detected for BD, RD, DAB, ADH, RP, SSC, Blush, FF, FT, TA, and pH. Furthermore, SNPs associated with more than one trait were identified on chromosome 1 (BD and TA; BD and FT; BD and FF; BD and Blush; DAB and Blush; and Blush, FT, and PW; FDIA and FW; FDIA and ADH; RD and SSC; and PW and TA), 2 (RD and PW; RD and pH; FF and FT; and FF and SSC), 3 (RD and DAB; DAB and SSC; Blush and RP; and FDIA and FW, ADH and SSC), 4 (BD and RD; RD and DAB; RD, DAB, FF, RP and FT; RD, DAB, FDIA, ADH, and RP; RD, DAB and Blush; RD, DAB and ADH; RD and FF; RD and ADH, RD, DAB and SSC; RD and SSC; RD, ADH and RP; RD, FF and SSC; RD, DAB, FDIA and RP; DAB and FF; DAB and FF; DAB, FF and RP; DAB, FT and PW, FDIA and FW; FDIA and RP; FDIA and ADH; FW, FF, ADH and RP; FF, ADH and FT; FDIA, FW, FF and RP; FF and FT; ADH and FT; FF, ADH and pH, ADH and PW, ADH and RP; and ADH and FT), 5 (BD and TA; RD and SSC; DAB and Blush, FDIA and FW; FW and SSC; FT, SSC, and TA; FT and PW; SSC and TA; FT and TA; SSC and pH; and TA and pH), 6 (RD and DAB; RD and FW; FDIA, FW, FT and SSC; FDIA and ADH; and FW and PW) and 8 (BD and pH; BD and SSC; BD and RD; BD and FF; RD, FDIA and FF; RD, DAB, PW and SSC; and FT and SSC).

The multi-locus model FarmCPU revealed a total of 180 QTNs (Supplementary Table 6). The highest number of QTNs were detected on chromosome 4 (33), while the smallest was observed on chromosome 7 (6). Consistently associated SNPs over at least two seasons were identified for BD, RD, ADH, RP, and SSC. In addition, SNPs associated with more than one trait were detected on chromosomes 1 (DAB and RD), 3 (Blush and RP; FW and RD), 4 (Blush and RD; RD and SSC; FF and SSC), 5 (TA and pH), and 8 (BD and RD; FT and SSC).

**FIGURE 1:**
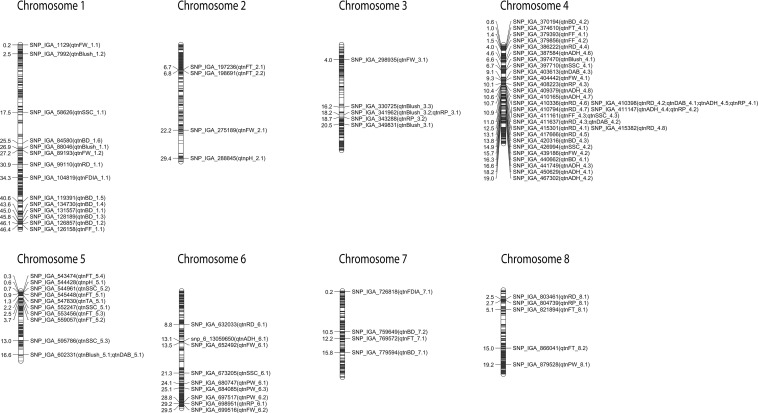
Reliable QTNs detected using different multi-locus GWAS methods and/or at least two seasons. Genetic distance scale in physical position (Mbp) is placed at left margin.

To ensure reliable results, further analyses included only QTNs that met the following conditions: QTNs detected in at least four methods in mrMLM and/or detected in at least two seasons using mrMLM (Table 2 and Figure 1); QTNs detected in two seasons using FarmCPU (Table 3 and Figure 1); and QTNs detected in at least three methods in mrMLM 4.0 and also identified in the FarmCPU (Table 4 and Figure 1).

**TABLE 2:** Significant associations between SNP markers and quality traits detected in at least three methods of mrMLM 4.0 and/or two seasons.

QTN	Trait	Method^*a*^	SNP	Chromosome	Position (bp)	LOD	*r*^2^ (%)
*qtnBD_1.1*	BD	1–4	SNP_IGA_131557	1	45022954	11.7 – 24.2	13.1 – 64.9
*qtnBD_1.2*	BD	1–4	SNP_IGA_126857	1	46125525	5.5 – 14.8	3.6 – 37.4
*qtnBD_1.3*	BD	1–4; 6	SNP_IGA_128189	1	45753343	3.7 – 11.1	1.3 – 6.3
*qtnBD_1.4*	BD	1–4	SNP_IGA_134730	1	43578596	4.9 – 9.4	0.4 – 2.4
*qtnBD_1.5*	BD	1,2,4,7	SNP_IGA_119391	1	40620294	4.0 – 5.9	1.4 – 8.5
*qtnBD_1.6*	BD	5; 7	SNP_IGA_84580	1	25541717	3.2 – 5.6	5.6 – 10.9
*qtnBD_4.1*	BD	2–5	SNP_IGA_440662	4	16306919	4.3 – 10.4	1.5 – 19.9
*qtnBD_7.1*	BD	1–3;5	SNP_IGA_779594	7	15842240	3.0 – 11.5	0.5 – 11.0
*qtnBD_7.2*	BD	4;6–7	SNP_IGA_759649	7	10525885	3.1– 4.6	1.1 – 28.7
*qtnRD_4.1*	RD	1–7	SNP_IGA_415301	4	12523245	3.5 – 12.8	0.8 – 37.2
*qtnRD_4.2*	RD	1–7	SNP_IGA_410398	4	10696489	4.3 – 43.0	5.7 – 36.3
*qtnRD_4.3*	RD	1–7	SNP_IGA_411637	4	10981971	5.7 –46.5	11.7 – 54.0
*qtnRD_4.4*	RD	1–4;6–7	SNP_IGA_386222	4	4045426	5.0 – 11.6	3.4 – 9.0
*qtnRD_4.5*	RD	1–4	SNP_IGA_417666	4	13091850	6.2 – 12.3	1.5 – 4.9
*qtnRD_4.6*	RD	1–3;7	SNP_IGA_410336	4	10676008	5.3 – 5.4	2.6 – 10.1
*qtnRD_4.7*	RD	1;6–7	SNP_IGA_410794	4	10890653	5.0 15.4	19.2 – 29.8
*qtnRD_6.1*	RD	1–2; 4–6	SNP_IGA_632033	6	8774913	5.1 – 10.1	1.6 – 6.6
*qtnDAB_4.1*	DAB	1–7	SNP_IGA_410398	4	10696489	5.2 – 38.4	7.9 – 28.4
*qtnDAB_4.2*	DAB	1–7	SNP_IGA_411637	4	10981971	5.4 – 56.6	16.9 – 56.1
*qtnDAB_4.3*	DAB	1–3; 5	SNP_IGA_403613	4	9052116	3.6 – 14.1	2.0 – 5.6
*qtnDAB_5.1*	DAB	1–3; 6	SNP_IGA_602331	5	16550893	5.7 – 10.0	13.0 – 20.3
*qtnBlush_1.1*	Blush	1–6	SNP_IGA_88046	1	26896332	3.0 – 15.0	3.7 – 24.4
*qtnBlush_1.2*	Blush	1–2; 7	SNP_IGA_7992	1	2518043	4.0 – 4.8	3.0 – 11.1
*qtnBlush_3.1*	Blush	1–2; 4–6	SNP_IGA_349831	3	20473077	3.1 – 5.2	1.5 – 8.0
*qtnBlush_3.2*	Blush	3; 6–7	SNP_IGA_341962	3	18179421	4.7 – 11.3	1.2 – 11.4
*qtnBlush_4.1*	Blush	2–4; 6	SNP_IGA_397470	4	6624729	3.8 – 5.7	12.1 – 26.1
*qtnBlush_5.1*	Blush	2–4; 6	SNP_IGA_602331	5	16550893	3.3 – 8.1	1.4 – 6.90
*qtnFDIA_7.1*	FDIA	1–2; 4; 6	SNP_IGA_726818	7	207697	3.0 – 5.1	4.8 – 7.9
*qtnFW_1.1*	FW	1–6	SNP_IGA_1129	1	209701	3.5 – 8.6	4.3 – 8.5
*qtnFW_1.2*	FW	1–2; 4–5	SNP_IGA_89193	1	27244316	4.7 – 10.4	8.5 – 19.4
*qtnFW_2.1*	FW	1–4; 6	SNP_IGA_275189	2	22195492	3.4 – 7.3	2.2 – 8.9
*qtnFW_3.1*	FW	1–2; 4; 6	SNP_IGA_298935	3	3989094	6.7 – 8.0	5.8 – 7.7
*qtnFW_4.1*	FW	1–2; 4; 6	SNP_IGA_404442	4	9321093	3.7 – 4.9	9.8 – 15.7
*qtnFW_4.2*	FW	1–6	SNP_IGA_439186	4	15742278	6.0 – 10.4	4.2 – 13.4
*qtnFW_6.1*	FW	1–6	SNP_IGA_652492	6	13508541	3.1 – 5.3	2.6 – 13.8
*qtnFW_6.2*	FW	1–2; 4–6	SNP_IGA_699516	6	29491714	4.7 – 6.5	5.6 – 7.7
*qtnFF_1.1*	FF	1–2; 4–5	SNP_IGA_126158	1	46430951	3.3 – 4.1	8.6 –16.4
*qtnFF_4.1*	FF	2–3; 5–6	SNP_IGA_379393	4	1391180	3.0 – 6.9	2.5 – 16.5
*qtnFF_4.2*	FF	2–5; 7	SNP_IGA_379856	4	1477791	3.3 – 7.9	1.7 – 4.0
*qtnFF_4.3*	FF	2–6	SNP_IGA_411161	4	10922075	4.3 – 11.2	2.2 – 9.8
*qtnADH_4.1*	ADH	1–6	SNP_IGA_450629	4	18235458	8.8 – 20.3	12.9 – 69.2
*qtnADH_4.2*	ADH	1–7	SNP_IGA_467302	4	19028425	6.3 – 69.0	5.3 – 30.4
*qtnADH_4.3*	ADH	1–2; 4–6	SNP_IGA_441749	4	16584598	4.1 – 12.4	2.5 –33.1
*qtnADH_4.4*	ADH	1–7	SNP_IGA_411147	4	10921604	3.8 – 13.0	2.2 – 29.6
*qtnADH_4.5*	ADH	1; 7	SNP_IGA_410398	4	10696489	4.6 – 7.2	2.8 – 6.5
*qtnADH_4.6*	ADH	1; 4; 6–7	SNP_IGA_387584	4	4601159	3.1 – 6.9	3.2 – 6.2
*qtnADH_4.7*	ADH	4; 6–7	SNP_IGA_410165	4	10641209	4.0 – 5.8	1.9 – 3.3
*qtnADH_6.1*	ADH	1–2; 4; 6	snp_6_13059650	6	13073956	4.1 – 8.5	4.7 – 10.2
*qtnRP_3.1*	RP	4; 6–7	SNP_IGA_341962	3	18179421	12.0 - 12.1	5.9 – 23.6
*qtnRP_3.2*	RP	1; 5; 7	SNP_IGA_343288	3	18666687	4.8 – 9.5	23.9 – 55.2
*qtnRP_4.1*	RP	1–7	SNP_IGA_410398	4	10696489	4.4 – 15.2	6.2 – 19.7
*qtnRP_4.2*	RP	1–2; 4; 6–7	SNP_IGA_411147	4	10921604	3.5 – 9.9	8.8 - 21.0
*qtnRP_4.3*	RP	4; 6–7	SNP_IGA_408223	4	10107085	3.3 – 4.2	1.2 – 4.1
*qtnRP_6.1*	RP	1–4; 6	SNP_IGA_698951	6	29242212	4.2 – 9.3	6.0 - 8.5
*qtnFT_2.1*	FT	3–4; 7	SNP_IGA_197236	2	6650921	3.3 – 7.2	2.1 – 4.9
*qtnFT_2.2*	FT	5–7	SNP_IGA_198691	2	6753400	4.9 – 7.5	5.2 – 16.4
*qtnFT_4.1*	FT	1–4	SNP_IGA_374610	4	994306	6.4 – 9.4	4.8 – 11.1
*qtnFT_5.1*	FT	1–3; 5	SNP_IGA_545448	5	850261	5.1 – 30.7	15.3 – 33.8
*qtnFT_5.2*	FT	1–2; 5–6	SNP_IGA_559057	5	3731800	7.9 –11.2	9.9 –16.7
*qtnFT_5.3*	FT	4; 6–7	SNP_IGA_553456	5	2477309	3.9 – 7.5	10.1 – 22.7
*qtnFT_7.1*	FT	1–7	SNP_IGA_769572	7	12248919	3.3 – 10.4	7.6 – 16.0
*qtnFT_8.1*	FT	1–4; 6	SNP_IGA_821894	8	5071328	3.5 – 6.8	3.4 – 6.4
*qtnFT_8.2*	FT	1–2; 4–5; 7	SNP_IGA_866041	8	15002010	3.7 – 4.2	0.9 – 4.2
*qtnPW_6.1*	PW	1–4; 6	SNP_IGA_680747	6	24132839	3.6 – 7.7	2.8 –5.7
*qtnPW_6.2*	PW	1–4; 6	SNP_IGA_697517	6	28795793	5.2 – 7.6	6.6 – 17.0
*qtnPW_6.3*	PW	1–2; 4–6	SNP_IGA_684085	6	25090090	3.7 – 9.1	8.4 – 28.6
*qtnPW_8.1*	PW	2; 4–6	SNP_IGA_879528	8	19234898	3.0 – 4.5	4.5 – 13.2
*qtnSSC_1.1*	SSC	1–4; 6	SNP_IGA_58626	1	17538855	12.6 – 25.0	1.6 – 17.4
*qtnSSC_4.1*	SSC	1–4	SNP_IGA_397710	4	6694626	11.8 – 15.0	12.3 – 16.4
*qtnSSC_4.2*	SSC	1–2; 4–6	SNP_IGA_426994	4	14898353	3.2 – 7.6	2.4 – 11.8
*qtnSSC_4.3*	SSC	1–2; 4–7	SNP_IGA_411161	4	10922075	4.2 – 13.4	0.8 – 7.5
*qtnSSC_5.1*	SSC	1–5	SNP_IGA_552247	5	2240224	8.7 – 31.4	5.3 – 24.0
*qtnSSC_5.2*	SSC	1–2; 4;6	SNP_IGA_544961	5	698215	4.2 – 7.1	3.3 – 8.4
*qtnSSC_5.3*	SSC	1–7	SNP_IGA_595786	5	13019899	3.2 – 7.4	1.1 – 5.6
*qtnSSC_6.1*	SSC	1–2; 4;6	SNP_IGA_673205	6	21277895	6.7 – 9.5	4.3 – 12.3
*qtnTA_5.1*	TA	1–2; 7	SNP_IGA_547830	5	1342919	3.1 – 20.4	1.0–38.7
*qtnpH_2.1*	pH	1–4; 6	SNP_IGA_288845	2	29376788	4.9 – 11.2	6.9 – 14.6
*qtnpH_5.1*	pH	1–2; 4–7	SNP_IGA_544428	5	557504	7.1 – 29.51	30.5 – 62.7

*BD, bloom date; RD, ripening date; DAB, days after bloom; FF, fruit diameter; FW, fruit weight; FF, fruit firmness; ADH, adherence; RP, redness around pit; FT, fruit texture; PW, pit weight; SSC, soluble solid concentration; TA, titratable acidity.*

*^*a*^Methods: 1 = mrMLM; 2 = FASTmrMLM; 3 = FASTmrEMMA; 4 = pLARmEB; 5 = pKWmEB; 6 = ISIS EM–BLASSO; 7 = detected in at least two seasons.*

**TABLE 3:** Significant associations between SNP markers and fruit quality traits consistently detected in two seasons using FarmCPU.

QTN	Trait/year	SNP	Chromosome	Position (bp)	*P*-value
*qtnBD_4.2*	BD_2011	SNP_IGA_370194	4	607467	1.37E-07
	BD_2012	SNP_IGA_370194	4	607467	7.74E-07
*qtnRD_4.7*	RD_2010	SNP_IGA_410794	4	10890653	2.58E-16
	RD_2011	SNP_IGA_410794	4	10890653	3.9E-39
	RD_2012	SNP_IGA_410794	4	10890653	1.47E-55
*qtnRD_4.8*	RD_2011	SNP_IGA_415382	4	12546297	1.2E-07
	RD_2012	SNP_IGA_415382	4	12546297	7.72E-11
*qtnRD_8.1*	RD_2011	SNP_IGA_803461	8	2534033	3.24E-13
	RD_2012	SNP_IGA_803461	8	2534033	1.59E-06
*qtnADH_4.8*	ADH_2010	SNP_IGA_409379	4	10389254	1.01E-06
	ADH_2011	SNP_IGA_409379	4	10389254	9.65E-09
*qtnADH_4.5*	ADH_2011	SNP_IGA_410398	4	10696489	8.03E-19
	ADH_2012	SNP_IGA_410398	4	10696489	5.49E-09
*qtnADH_4.2*	ADH_2011	SNP_IGA_467302	4	19028425	8.31E-14
	ADH_2012	SNP_IGA_467302	4	19028425	4.8E-10
*qtnRP_3.1*	RP_2010	SNP_IGA_341962	3	18179421	6.64E-11
	RP_2011	SNP_IGA_341962	3	18179421	2.93E-16
*qtnSSC_5.3*	SSC_2011	SNP_IGA_595786	5	13019899	2.87E-08
	SSC_2012	SNP_IGA_595786	5	13019899	1.89E-06
*qtnSSC_6.1*	SSC_2011	SNP_IGA_673205	6	21277895	1.73E-06
	SSC_2012	SNP_IGA_673205	6	21277895	2.42E-06

*BD, bloom date; RD, ripening date; ADH, adherence; RP, red in pit; SSC, soluble solid concentration.*

**TABLE 4:** Significant associations between SNP markers and quality traits commonly detected using at least three mrMLM 4.0 GWAS methods and FarmCPU.

QTN	Trait	SNP	Chromosome	Position (bp)
*qtnBD_1.1*	BD	SNP_IGA_131557	1	45022954
*qtnBD_1.2*	BD	SNP_IGA_126857	1	46125525
*qtnBD_1.3*	BD	SNP_IGA_128189	1	45753343
*qtnBD_4.3*	BD	SNP_IGA_420316	4	13813285
*qtnRD_1.1*	RD	SNP_IGA_99110	1	30864365
*qtnRD_4.1*	RD	SNP_IGA_415301	4	12523245
*qtnRD_4.2*	RD	SNP_IGA_410398	4	10696489
*qtnRD_4.5*	RD	SNP_IGA_417666	4	13091850
*qtnDAB_4.2*	DAB	SNP_IGA_411637	4	10981971
*qtnBlush_3.3*	Blush	SNP_IGA_330725	3	16198112
*qtnBlush_5.1*	Blush	SNP_IGA_602331	5	16550893
*qtnFDIA_1.1*	FDIA	SNP_IGA_104819	1	34325189
*qtnFW_6.1*	FW	SNP_IGA_652492	6	13508541
*qtnFF_4.1*	FF	SNP_IGA_379393	4	1391180
*qtnFF_4.3*	FF	SNP_IGA_411161	4	10922075
*qtnADH_4.1*	ADH	SNP_IGA_450629	4	18235458
*qtnADH_4.2*	ADH	SNP_IGA_467302	4	19028425
*qtnADH_4.3*	ADH	SNP_IGA_441749	4	16584598
*qtnRP_4.2*	RP	SNP_IGA_411147	4	10921604
*qtnRP_6.1*	RP	SNP_IGA_698951	6	29242212
*qtnRP_8.1*	RP	SNP_IGA_804739	8	2702428
*qtnFT_5.1*	FT	SNP_IGA_545448	5	850261
*qtnFT_5.4*	FT	SNP_IGA_543474	5	329318
*qtnSSC_1.1*	SSC	SNP_IGA_58626	1	17538855
*qtnSSC_4.1*	SSC	SNP_IGA_397710	4	6694626
*qtnSSC_4.2*	SSC	SNP_IGA_426994	4	14898353
*qtnSSC_4.3*	SSC	SNP_IGA_411161	4	10922075
*qtnSSC_5.1*	SSC	SNP_IGA_552247	5	2240224
*qtnSSC_5.3*	SSC	SNP_IGA_595786	5	13019899
*qtnpH_2.1*	pH	SNP_IGA_288845	2	29376788
*qtnpH_5.1*	pH	SNP_IGA_544428	5	557504

*BD, bloom date; RD, ripening date; DAB, days after bloom; FDIA, fruit diameter; FW, fruit weight; FF, fruit firmness; ADH, adherence; RP, redness around pit; FT, fruit texture; SSC, soluble solid concentration.*

### QTNs Detected With Four Methods and/or Two Seasons Using mrMLM R Package

The mrMLM revealed nine reliable QTNs distributed on 3 chromosomes significantly associated with BD (Table 2 and Figure 1). The *qtnBD_1.1*, *qtnBD_1.2*, *qtnBD_1.3*, *qtnBD_1.4*, *qtnBD_1.5*, and *qtnBD_1.6*, on chromosome 1, explained 13.1–64.9, 3.6–37.4, 1.3–6.3, 0.4–2.4, 1.4–8.5, and 5.6–10.9% of total phenotypic variation, respectively. The *qtnBD_4.1* located on chromosome 4, demonstrated LOD score of 4.3–10.4 and explained 1.5–19.8% of phenotypic variation. The reliable QTNs identified on chromosome 7 (*qtnBD_7.1* and *qtnBD_7.2*) explained 0.5–11.1 and 1.1–28.7% of total phenotypic variation, respectively. In addition, the *qtnBD_1.5*, *qtnBD_1.6* and *qtnBD_7.2* were detected in two seasons.

Reliable QTNs associated with RD were detected on chromosomes 4 (*qtnRD_4.1*, *qtnRD_4.2*, *qtnRD_4.3*, *qtnRD_4.4*, *qtnRD_4.5*, *qtnRD_4.6* and *qtnRD_4.7*) and 6 (*qtnRD_6.1*). The *qtnRD_4.3* accounted for the highest phenotypic variation (11.7–54.0%) in comparison to the other QTNs identified on chromosome 4 (Table 2 and Figure 1). The *qtnRD_6.1*, on chromosome 6 demonstrated LD scores ranging from 5.1 to 10.1 and explained 1.6–6.6% of phenotypic variation. Almost all QTNs detected on chromosome 4 were detected in at least two seasons, except *qtnRD_4.5.*

Three QTNs on chromosome 4 (*qtnDAB_4.1*, *qtnDAB_4.2* and *qtnDAB_4.3*) were associated with DAB. The greatest phenotypic variation was explained by *qtnDAB_4.2* (16.9–56.1%). The *qtnDAB_5.1* on chromosome 5 explained 13.0 to 20.3% of the phenotypic variance observed (LOD scores 5.7–10.0). The *qtnDAB_4.1* and *qtnDAB_4.2* on chromosome 4 were detected in two seasons.

The reliable QTNs associated with blush were distributed on chromosomes 1 (*qtnBlush_1.1* and *qtnBlush_1.2*), 3 (*qtnBlush_3.1* and *qtnBlush_3.2*), 4 (*qtnBlush_4.1*), and 5 (*qtnBlush_5.1*). The *qtnBlush_3.2* was identified in three seasons, while the *qtnBlush_1.2* was identified in two. The *qtnBlush_4.1* explained the highest phenotypic variation (12.1–26.1%).

The *qtnFDIA_7.1* associated with FDIA was located on chromosome 7, revealed LOD scores ranging from 3.0 to 5.1 and explained 4.8–7.9% of the phenotypic variance observed.

Concerning FW, QTNs were detected on chromosomes 1 (*qtnFW_1.1* and *qtnFW_1.2*), 2 (*qtnFW_2.1*), 3 (*qtnFW*_3.1), 4 (*qtnFW_4.1* and *qtnFW_4.2*), and 6 (*qtnFW_6.1* and *qtnFW_6.2*). The *qtnFW_1.1*, *qtnFW_4.2* and *qtnFW_6.1* were detected with the six methods of the mrMLM R package. The greatest phenotypic variation was explained by *qtnFW_1.2* (8.5–19.4%), followed by *qtnFW_4.1* (9.8–15.7%).

Quantitative trait nucleotides associated with FF were located on chromosomes 1 (*qtnFF_1.1*) and 4 (*qtnFF_4.1*, *qtnFF_4.2* and *qtnFF_4.3).* The *qtnFF_4.2* was identified in two seasons and explained 1.7–4.0% of the phenotypic variation with the *qtnFF_1.1* explaining the highest phenotypic variation for FF (8.6–16.4%).

For ADH, seven QTNs on chromosome 4 (*qtnADH_4.1*, *qtnADH_4.2*, *qtnADH_4.3*, *qtnADH_4.4*, *qtnADH_4.5*, *qtnADH_4.6* and *qtnADH_4.7*), and one on chromosome 6 (*qtnADH_6.1)* were identified. The *qtnADH_4.2* was detected in three seasons, while *qtnADH_4.4*, *qtnADH_4.5*, *qtnADH_4.6* and *qtnADH_4.7* were detected in two seasons. The *qtnADH_4.1* was identified with the six methods of mrMLM and explained the highest phenotypic variance (12.9–69.2%).

Quantitative trait nucleotides on chromosome 3 (*qtnRP_3.1* and *qtnRP_3.2*), 4 (*qtnRP_4.1*, *qtnRP_4.2* and *qtnRP_4.3*), and 6 (*qtnRP_6.1*) were significantly associated with RP. The *qtnRP_4.1* was detected in three seasons, while the *qtnRP_3.1*, *qtnRP_3.2, qtnRP_4.2* and *qtnRP_4.3* were detected in two seasons. The highest phenotypic variation (23.9–55.2%) was explained by the *qtnRP_3.2* located on the chromosome 3.

The reliable QTNs associated with FT were distributed on chromosome 2 (*qtnFT_2.1* and *qtnFT_2.2*), 4 (*qtnFT_4.1*), 5 (*qtnFT_5.1*, *qtnFT_5.2* and *qtnFT_5.3*), 7 (*qtnFT_7.1*) and 8 (*qtnFT_8.1* and *qtnFT_8.2*). The *qtnFT_2.1*, *qtnFT_2.2*, *qtnFT_5.3*, *qtnFT_7.1* and *qtnFT_8.2* were detected in two seasons. The *qtnFT_5.1* and *qtnFT_5.3* exhibited the highest phenotypic variation 15.3–33.8 and 10.1–22.7%, respectively.

Quantitative trait nucleotides associated with PW were identified on chromosomes 6 (*qtnPW_6.1*, *qtnPW_6.2* and *qtnPW_6.3*) and 8 (*qtnPW_8.1)*. The *qtnPW_6.2* and *qtnPW_6.3* with LOD scores of 5.2–7.6 and 3.7–9.1 explained 6.6–17.0 and 8.4–28.6% of the phenotypic variation, respectively.

For SSC, 8 reliable QTNs were detected on chromosomes 1 (*qtnSSC_1.1*), 4 (*qtnSSC_4.1*, *qtnSSC_4.2* and *qtnSSC_4.3)*, 5 (*qtnSSC_5.1*, *qtnSSC_5.2* and *qtnSSC_5.3)*, and 6 (*qtnSSC_6.1*), with the *qtnSSC_4.3* and *qtnSSC_5.3* detected in two seasons. The *qtnSSC_4.1* and *qtnSSC_5.1* accounted for the highest phenotypic variation (12.3–16.4 and 5.3–24.0%).

The *qtnTA_5.1* on chromosome 5 was associated with TA and detected in two seasons. The LOD score varied from 3.1 to 20.4 and explained 1.0–38.0% of the phenotypic variation.

One reliable QTN associated with pH was identified on chromosome 2 (*qtnpH_2.1*) and chromosome 5 (*qtnpH_5.1*). The *qtnpH_5.1* explained the highest phenotypic variation (30.5–62.7%) and was detected in two seasons.

### QTNs Detected in Two Seasons Using FarmCPU

The *qtnBD_4.2* was consistently associated with BD in the two seasons and revealed a *p* values of 1.37E-07 and 7.74E-07, respectively (Table 3 and Figure 1).

The *qtnRD_4.7* located on chromosome 4 (10.9 Mbp) was significantly associated with RD in all three seasons. In addition, the *qtnRD_4.8* and *qtnRD_8.1* were associated with RD in 2011 and 2012 and were located at 12.5 Mbp (chromosome 4) and 2.5 Mbp (chromosome 8), respectively.

A reliable *qtnADH_4.8* on chromosome 4 (10.4 Mb) was consistently associated with ADH in 2010 and 2011, while *qtnADH_4.5* (10.69) and *qtnADH_4.2* (19.02 Mbp) were associated with ADH in 2011 and 2012.

For RP, the *qtnRP_3.1* located at 18.17 Mbp on chromosome 3 was detected in two seasons with *p* values of 6.64E-11 and 2.93E-16, respectively.

Consistent, significant associations with the SSC were detected in two seasons with QTNs on chromosomes 5 (*qtnSSC_5.3*) and 6 (*qtnSSC_6.1*).

### QTNs Commonly Detected With Both mrMLM and FarmCPU

A total of 31 QTNs were consistently detected with at least three methods in mrMLM and also with the FarmCPU (Table 4). Six QTNs associated with BD, RD, FDIA, and SSC were identified on chromosome 1 (*qtnBD_1.1*, *qtnBD_1.2*, *qtnBD_1.3*, *qtnRD_1.1*, *qtnFDIA_1.1*, and *qtnSSC_1.1*). Only one QTN (*qtnpH_2.1*) associated with pH, located at 29.4 Mpb on chromosome 2 was detected using both approaches. A QTN associated with blush was detected on chromosome 3 (16.2 Mbp). Most of the QTNs were observed on chromosome 4. A total of twelve QTNs associated with BD (*qtnBD_4.2*), RD (*qtnRD_4.1*, *qtnRD_4.2* and *qtnRD_4.5*), DAB (*qtnDAB_4.2*), FF (*qtnFF_4.1* and *qtnFF_4.3*), ADH (*qtnADH_4.1*, *qtnADH_4.2* and *qtnADH_4.3*), RP (*qtnRP_4.2*) and SSC (*qtnSSC_4.1* and *qtnSSC_4.2*) and a QTN cluster of FF and SSC (*qtnFF_4.3* and *qtnSSC_4.3*) were identified. QTNs for blush (*qtnBlush_5.1*), FT (*qtnFT_5.1* and *qtnFT_5.4*), SSC (*qtnSSC_5.1* and *qtnSSC_5.3*) and pH (*qtnpH_5.1*) were identified on chromosome 5 located at 16.55, 0.85, 0.32, 2.24, 13.01 and 0.55 Mbp, respectively. Two QTNs associated with FW (*qtnFW_6.1*) and RP (*qtnRP_6.1*) were present on chromosome 6. Lastly, the QTN *qtnRP_8.1*, associated with the RP, was present on chromosome 8 (2.7 Mbp).

### Candidate Genes

Genomic regions encompassing the QTNs detected using the mrMLM 4.0 methods and FarmCPU revealed a total of twenty-eight haploblocks located at scaffolds 1, 2, 3, 4, 5, 6, and 8 (Table 5).

**TABLE 5:** Haploblock regions encompassing SNPs markers significantly associated with fruit quality traits in peach.

Trait	Hap	Scaffold	Start (bp)	End (bp)	Associated SNPs
SSC	1_1	1	17400346	17538855	SNP_IGA_58626
RD	1_2	1	30644296	31160594	SNP_IGA_99110
FDIA	1_3	1	34122904	34404100	SNP_IGA_104819
BD	1_4	1	44904968	45237616	SNP_IGA_131557
BD	1_5	1	45753343	45821173	SNP_IGA_128189
BD	1_6	1	46012310	46430951	SNP_IGA_126857
pH	2_1	2	29241773	29376788	SNP_IGA_288845
Blush	3_1	3	16195795	16236799	SNP_IGA_330725
FF	4_1	4	1382161	1413701	SNP_IGA_379393
SSC	4_2	4	6688718	6712809	SNP_IGA_397710
RD	4_3	4	10676008	10760085	SNP_IGA_410398
RP	4_4	4	10760086	10981971	SNP_IGA_411147
FF	4_4	4	10760086	10981971	SNP_IGA_411161
SSC	4_4	4	10760086	10981971	SNP_IGA_411161
DAB	4_4	4	10760086	10981971	SNP_IGA_411637
RD	4_5	4	12429145	12523245	SNP_IGA_415301
RD	4_6	4	13078233	13108512	SNP_IGA_417666
BD	4_7	4	13561808	14018643	SNP_IGA_420316
SSC	4_8	4	14735598	15182577	SNP_IGA_426994
ADH	4_9	4	16511312	16674024	SNP_IGA_441749
ADH	4_10	4	18140428	18235458	SNP_IGA_450629
ADH	4_11	4	18719887	19206580	SNP_IGA_467302
FT	5_1	5	329318	481015	SNP_IGA_543474
pH	5_2	5	521865	821356	SNP_IGA_544428
FT	5_3	5	850261	882334	SNP_IGA_545448
SSC	5_4	5	2086499	2242971	SNP_IGA_552247
SSC	5_5	5	13014155	13019899	SNP_IGA_595786
Blush	5_6	5	16550893	16702088	SNP_IGA_602331
FW	6_1	6	13235506	13565811	SNP_IGA_652492
RP	6_2	6	29231386	29714220	SNP_IGA_698951
RP	8_1	8	2369263	2838462	SNP_IGA_804739

*BD, bloom date; RD, ripening date; DAB, days after bloom; FDIA, fruit diameter; FW, fruit weight; FF, fruit firmness; ADH, adherence; RP, red in pit; FT, fruit texture; SSC, soluble solid concentration.*

**FIGURE 2:**
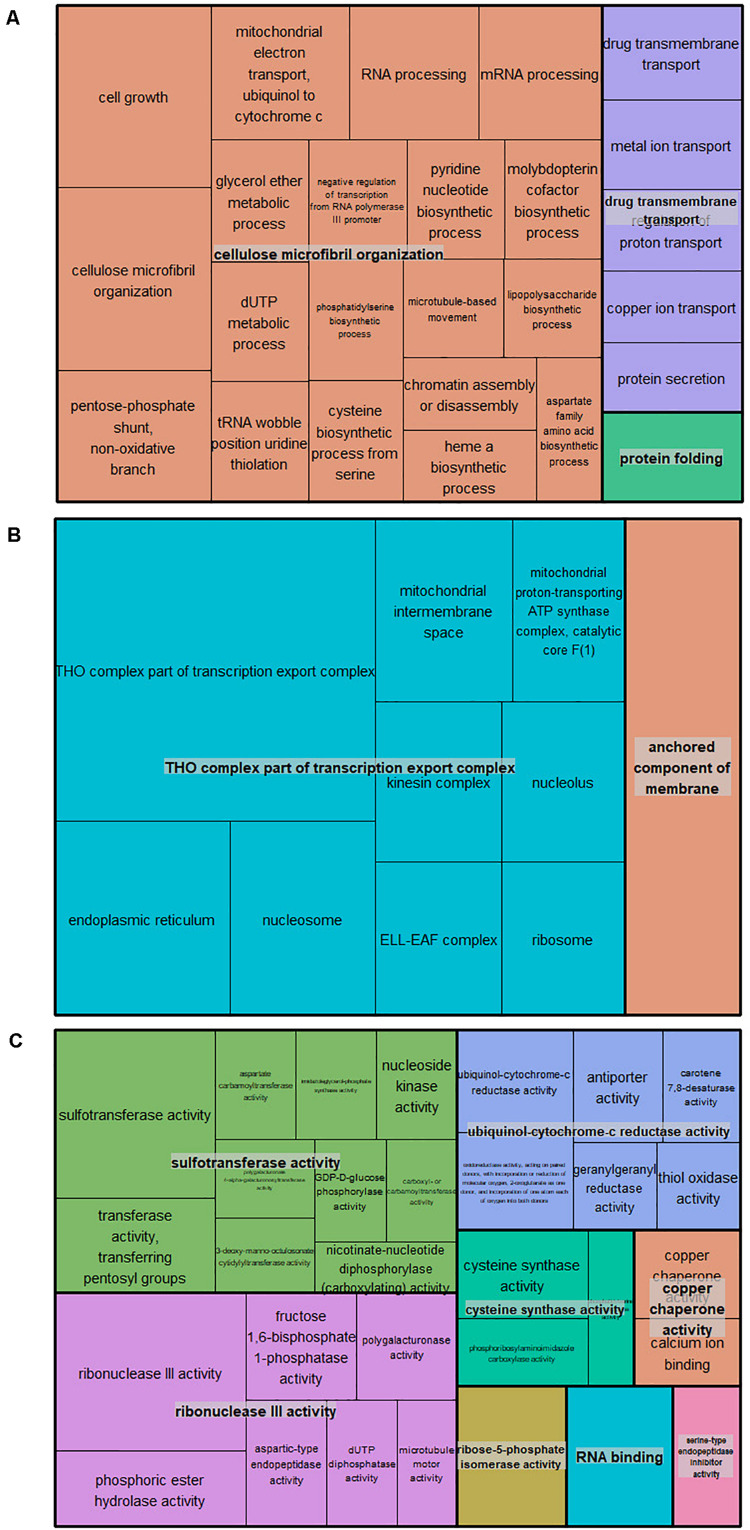
TreeMap of the GO term cluster representatives joined into “superclusters” of biological processes **(A)**, cellular component **(B)**, and molecular functions **(C)** visualized with different colors. Each rectangle is a single cluster representative.

A total of 566 candidate genes (CG) were detected within the haploblock regions for the significantly associated QTNs (Supplementary Table 7), from which 93 CG were detected in the regions for BD, 89 for RD, 29 for DAB, 22 for Blush, 39 for FDIA, 24 for FW, 26 for ADH, 31 for FF, 148 for RP, 12 for FT and 90 for SSC. The gene ontology (GO) annotations were retrieved for 435 CG. The GO enrichment analysis revealed 68 GO terms in all three GO aspects, biological process, molecular function, and cellular component. Twenty-six GO terms (78 genes) were described as biological processes, 32 GO terms (108 genes) with the molecular function, and 10 GO terms (36 genes) with the cellular component (Supplementary Table 8). The GO term cluster representatives were joined into “superclusters” of terms loosely related to cellulose microfibril organization, THO complex part of transcription export complex and sulfotransferase activity in the biological process, cellular component and molecular function, respectively (Figure 2).

### Hotspots in Peach Genome

The reliable QTNs revealed fruit quality hotspots in the peach genome (Figure 1). On chromosome 1, three reliable QTNs associated with BD, Blush and FW in the interval of 25.5–27.2 Mbp were identified. In addition, at the bottom of the same chromosome (43.6–46.4 Mbp), we also observed QTNs associated with BD and FF. On chromosome 3, a hotspot involving the quality traits Blush and RP was observed in the region located at 18.2–20.2 Mbp. The majority of the reliable QTNs detected were located on chromosome 4 (0.6–19.0 Mbp), especially concentrated in the genomic region located at 9.0–12.5 Mbp with QTNs associated with DAB, FW, RP, ADH, RD, FF, and SSC. A hotspot was also observed on the top of chromosome 5 (0.3–3.7 Mbp) with significant signals associated with FT, pH, SSC, and TA. In the genomic region on chromosome 6 spanning 28.8–29.5 Mbp, QTNs involving PW, RP, and FW were detected. Furthermore, on top of chromosome 8 (2.5–5.1 Mbp), a hotspot with reliable QTNs associated with RD, RP, and FT was observed.

## Discussion

We have analyzed peach germplasm containing 620 individuals from three U.S. public fresh market breeding programs [University of Arkansas System Division of Agriculture (AR), Clemson University (SC) and Texas A&M University (TX)] for 14 traits over three seasons (2010, 2011, and 2012). Phenotypic variation was observed between individuals and seasons, and the mean values for BD, RD, FW, and SSC were lower than those reported in the Spanish and European germplasm (Hernández Mora et al., 2017; Font I Forcada et al., 2019). However, average values for RD and DAB observed in our study were in agreement with the values reported in the University of Guelph’s peach germplasm, comprised of accessions originating from different regions across North America (Elsadr et al., 2019). A high and significant correlation between FW and FDIA (0.92) was previously observed in peach (da Silva Linge et al., 2015; Abdelghafar et al., 2020), as well as the positive correlation between RD and DAB (Elsadr et al., 2019) and the negative correlation between TA and pH (Abidi et al., 2011). In addition, the high estimated narrow sense heritability coefficients observed in this study ranging from 0.68 to 0.87, suggesting that the phenotypic variations of all traits are mainly affected by genetic factors, and therefore this dataset can be used for further genetic analyses.

The mean observed heterozygosity (Ho = 0.36) in the U.S. peach germplasm was greater than that observed in the germplasm from four European, one Chinese and one Brazilian peach collections reported in previous studies (Micheletti et al., 2015; Thurow et al., 2019). In addition, the mean inbreeding coefficient of 0.05 indicated a low level of inbreeding. The low mean of the inbreeding coefficient observed in this study could be attributed to the diverse material, including F1 and F2 populations with different genetic backgrounds.

The multidimensional scaling (MDS) clustered material into two groups, a group of individuals from TX fresh market breeding program related to ‘Tropic Beauty’, ‘TX2293_3’, ‘TX2B136’, ‘TXW1293_1’ and ‘TXW1490_1’, and the second group, comprised of individuals from the AR and SC fresh market breeding programs. Breeding material from AR and SC clustered in one main group, due to the common founders or pedigree-linkages of some breeding populations. Nevertheless, the second group could further be separated in two clusters in which the first cluster grouped the individuals linked to ‘A_663’, ‘A_760’, and ‘Bolinha’, and the second cluster contained individuals linked to ‘Clayton’ and/or ‘O’Henry’. The population structure indicated by fastSTRUCTURE, between *K* = 2 and 19, supported MDS clustering, as the *K* = 3 reflected grouping based on the pedigree background, and number of the breeding programs in the panel.

The population structure influences LD patterns within the genome (Thurow et al., 2019). The LD detected in this study decayed much slower in comparison with the observed by Thurow et al. (2019) and faster than the observed by Micheletti et al. (2015). The difference could be explained by the genetic material analyzed and the methods used for analyzing the LD decay. In addition, a slower decay of LD is expected in selfing materials. The LD decay over distance also determines the number of markers required to cover the genome. Considering the LD decay of our dataset (540 Kb), approximately 421 SNPs covering the total peach genome (227.4 Mb) should be sufficient to perform the GWAS. However, domestication regions containing key genes require more SNPs due to the faster LD decay (Cao et al., 2016).

### Multi-Locus GWAS

In order to control the false positive rate in GWAS analysis, conservative correction methods such as false discovery rate (FDR) and Bonferroni correction are frequently adopted in association studies. However, these corrections are often too conservative for detecting many important loci. Thus, multi-locus GWAS methods have been recommended to overcome the problem of stringent correction (Zhang et al., 2020). In this study, we have successfully performed a genome-wide association study using six multi-locus GWAS methods (mrMLM, FASTmrMLM, FASTmrEMMA, pLARmEB, pKWmEB, and ISIS EM-BLASSO) comprised in mrMLM 4.0 and FarmCPU R packages. The mrMLM 4.0 adopts the critical probability value or log of odds (LOD), a less stringent significance threshold while FarmCPU requires Bonferroni correction to detect QTNs. The multi-locus methods detected 967 and 180 QTNs using mrMLM 4.0 and FarmCPU, respectively, allowing the identification of important regions in the peach genome that control fruit quality traits. Furthermore, consistently reliable QTNs (88) for all traits were detected using different multi-locus GWAS methods and/or at least two seasons (Tables 2–4). Half of the reliable QTNs (44) detected have already been reported using different progenies, germplasm and approaches. However, to our knowledge, the other 44 reliable QTNs controlling fruit quality traits in peach have not been previously described.

One of the main goals of breeding programs is the development of commercial varieties with predictable bloom time to adapt to various target environments. Therefore, understanding the genetic architecture of phenology-related traits represents a key prerequisite to enable the development of varieties adapted to different climates (Gogorcena et al., 2020). Reliable QTNs associated with bloom date (BD), the *qtnBD_1.1*, *qtnBD_1.2*, *qtnBD_1.3*, *qtnBD_1.4*, *qtnBD_4.1*, *qtnBD_4.3*, *qtnBD_7.1* collocate near or in the same regions previously reported using QTL mapping and pedigree-based analysis (PBA) (Fan et al., 2010; Romeu et al., 2014; Bielenberg et al., 2015; Hernández Mora et al., 2017; Rawandoozi et al., 2020a). The fact that these regions were identified following different approaches (linkage analysis, PBA analysis and GWAS) in diverse genetic material, makes them an interesting source of allelic variation for BD in peach.

QTL mapping and association studies focused on ripening date have been widely reported in peach (Eduardo et al., 2011; Pirona et al., 2013; Fresnedo-Ramírez et al., 2015; Hernández Mora et al., 2017; Elsadr et al., 2019; Font I Forcada et al., 2019; Nuñez-Lillo et al., 2019; Rawandoozi et al., 2020a). The *qtnRD_4.2*, *qtnRD_4.3*, *qtnRD_4.6* and *qtnRD_4.7* overlapped with the major RD QTLs reported in the chromosome 4 located at approximately 10.6 and 11.1 Mbp (Pirona et al., 2013; Elsadr et al., 2019). In addition, the *qtnRD_4.1*, *qtnRD_4.5* and *qtnRD_4.8* were located in the same genetic interval (11.2 - 14.1 Mbp) of the RD QTL reported by Hernández Mora et al. (2017) using the pedigree-based QTL mapping in the European peach germplasm. Beside chromosome 4, we also detected reliable QTNs for RD on chromosome 1 (*qtnRD_1.1*), 6 (*qtnRD_6.1*) and 8 (*qtnRD_8.1*). RD QTLs and associated SNPs on chromosome 1 were previously reported in peach at approximately 12.0 Mbp (Fresnedo-Ramírez et al., 2015), 35 Mbp (Romeu et al., 2014; Font I Forcada et al., 2019), and 40.0–47.0 Mbp (Romeu et al., 2014; Hernández Mora et al., 2017; Font I Forcada et al., 2019; Nuñez-Lillo et al., 2019). The reliable *qtnRD_1.1* detected in this study using mrMLM and FarmCPU was located at 30.9 Mbp. Moreover, the *qtnRD_6.1* (chr6: 8.8 Mb) and *qtnRD_8.1* (chr8: 2.5 Mbp) were in close proximity of the SNPs associated with RD detected on chromosomes 6 (SNP_IGA_630302; 8.3 Mbp) and 8 (SNP_IGA_806528; 2.9 Mbp) in the Spanish germplasm (Font I Forcada et al., 2019).

QTL clusters for RD and DAB were commonly detected in peach (Fresnedo-Ramírez et al., 2015; Hernández Mora et al., 2017; Elsadr et al., 2019; Rawandoozi et al., 2020a). In this study, two reliable QTNs (*qtnDAB_4.1* and *qtnDAB_4.2*) associated with DAB overlapped with the QTNs associated with RD. The position of the *qtnDAB_4.1* (chr 4: 10.7 Mbp) matched the associated SNPs identified in a panel of 132 peach accessions genotypically characterized via genotyping by sequencing (GBS) approach (Elsadr et al., 2019). In addition, the SNP_IGA_410398 was emphasized as a predictive SNP for RD and DAB in haplotype analysis in a DAB QTL detected using PBA approach (Rawandoozi et al., 2020a). The *qtnDAB_4.2* (10.9 Mbp) was close to QTL for DAB detected using PBA in the European germplasm (Hernández Mora et al., 2017). Thus, our results confirmed the location of RD and DAB associated regions in the peach genome, and due to their importance for breeding, could be useful in selection of various phenology patterns in future studies.

Previous QTL analyses in peach have identified a major QTL for blush on chromosome 3 accounting, in average, for 63.7% of observed phenotypic variation (Frett et al., 2014). The *qtnBlush_3.1, qtnBlush_3.2* and *qtnBlush_3.3* were located within the genetic interval of the major QTL for blush on chromosome 3. QTL regions for blush on chromosome 4 were mapped approximately at 10.5 – 11.5 Mbp (Rawandoozi et al., 2020b); 11.2 – 14.1 Mbp (Hernández Mora et al., 2017); and at 19.8 and 28.6 Mb (Shi et al., 2020). The *qtnBlush_4.1* was detected at 6.6 Mbp and accounted for the highest phenotypic variation observed. Moreover, we identified two QTNs on chromosome 1 (*qtnBlush_1.1:* 26.9 Mbp; *qtnBlush_1.2:* 2.5 Mbp). Analyzing an F1 peach population derived from the cross between “Shahong” and “Hongfurong,” Shi et al. (2020) also observed a QTL associated with blush on chromosome 1. However, the genetic interval was approximately at 21.5 Mbp. Although the percentage of the phenotypic variation explained was low, a QTN associated with blush (*qtnBlush_5.1*) on chromosome 5 was also detected.

Understanding the genetic control of fruit diameter and weight is an important goal of breeding programs due to the importance of these traits for the fresh market (Yue et al., 2014). QTL regions associated with fruit diameter and weight have been detected in all chromosomes (da Silva Linge et al., 2015; Fresnedo-Ramírez et al., 2016; Zeballos et al., 2016; Hernández Mora et al., 2017; Cao et al., 2019; Abdelghafar et al., 2020; Shi et al., 2020). In this study, we identified two reliable QTNs associated with FDIA (*qtnFDIA_7.1* and *qtnFDIA_1.1*). The *qtnFDIA_7.1*, on chromosome 7, is in the vicinity to fruit width and fruit depth QTLs (qP-Fwd7.2 and qP-Fd7.2) reported by da Silva Linge et al. (2015) using F_2_ progeny resulting from a cross between an ornamental peach PI91459 (“NJ Weeping”) × “Bounty.” The *qtnFDIA_1.1* (36.9 Mbp) was identified in a different region of chromosome 1 when compared with previous linkage analyses, where QTLs were located approximately at 11 Mbp (da Silva Linge et al., 2015); 27–28 Mbp (Hernández Mora et al., 2017); 41 Mbp (Zeballos et al., 2016) and 43 Mbp (da Silva Linge et al., 2015; Abdelghafar et al., 2020). Concerning position of *qtnFW_1.2* and *qtnFW_6.2* matched the QTL interval identified in European peach germplasm (Hernández Mora et al., 2017). The FW QTN *qtnFW_2.1* and *qtnFW_3.1* could be the FW QTLs mapped in an interspecific cross between peach and a wild relative *Prunus davidiana* (Quilot et al., 2004; Desnoues et al., 2016). On chromosome 4 *qtnFW_4.1* was close to the FW QTL reported by da Silva Linge et al. (2015) and *qtnFW_4.2* was in the same genetic interval of the QTL identified by Shi et al. (2020).

Fruit firmness (FF) represents an essential indicator of fruit quality for peach consumers. For this reason, several authors have investigated the genetic mechanisms controlling this trait in peach (Peace et al., 2005; Eduardo et al., 2011, 2015; Martínez-García et al., 2013; Nuñez-Lillo et al., 2015; Zeballos et al., 2016; Serra et al., 2017; Carrasco-Valenzuela et al., 2019). The *qtnFF_4.1* and *qtnFF_4.2*, reported in this study, were located in chromosome 4 with the position matching the QTL interval associated with firmness loss mapped by Serra et al. (2017). Moreover, the *qtnFF_4.3* was in the same genetic region in which Carrasco-Valenzuela et al. (2019) detected a QTL significantly associated with softening rate and Zeballos et al. (2016) detected a QTL for fruit firmness.

Flesh adherence to the pit (ADH) is another factor determining overall peach fruit quality, with consumers preferring freestone or semi freestone characteristics (Olmstead et al., 2015). Previous studies have shown that ADH is inherited and controlled by the Freestone-Melting (*F-M*) locus on chromosome 4, with genes encoding endopolygalacturonase (endoPG) associated with this trait (Peace et al., 2005; Gu et al., 2016). We detected *qtnADH_4.1* and *qtnADH_4.2* explaining the majority of the phenotypic variation close to the genetic region where the endoPG gene is located. Similar to Shi et al. (2020), we detected significant genetic regions associated to ADH in different regions of chromosome 4 and also in other chromosomes.

The QTNs associated with redness around the pit (RP) (*qtnRP_3.1* and *qtnRP_3.2*) located approximately at 18.2 and 18.7 Mb, on chromosome 3, matched the position of the associated signals to flesh color around the stone detected in the recent GWAS using genome structural variations (SVs) (Guo et al., 2020). In addition, the *Cs* locus associated with red color around the pit was previously mapped in the middle of chromosome 3 (Yamamoto et al., 2001). Interestingly, the SNP_IGA_341962 (*qtnRP_3.1*) was also associated with blush (*qtnBlush_3.2*). Therefore, the QTNs associated with RP identified on chromosome 4 (*qtnRP_4.1*, *qtnRP_4.2* and *qtnRP_4.3*) were close to the associated signals detected by Guo et al. (2020) and in a different region of the associated SNPs reported by Cao et al. (2016), while the QTNs detected on chromosome 6 (*qtnRP_6.1*) and 8 (*qtnRP_8.1*) were located in different regions when compared with previous studies (Cao et al., 2016; Guo et al., 2020).

Concerning pit weight (PW), the *qtnPW_6.1*, identified on chromosome 6, is close to the QTL (qSW6; 24.6 Mb) mapped in the interspecific cross between almond × peach population (Donoso et al., 2016). Cao et al. (2016) also detected a region significantly associated with PW on chromosome 6; however, the location was approximately at 26.9 Mbp.

Soluble solid concentration is one of the most important quality traits in peach, with consumers expecting an enhanced sugar content or sweetness perception for the low acid types (Cirilli et al., 2016). Therefore, SSC has been a target trait in several studies involving intra- and interspecific progenies and germplasm to access the genetic potential and consequently improve the sugar content in new cultivars. We detected QTNs associated with SSC on chromosome 1, 4, 5, and 6. The *qtnSSC_5.1* on chromosome 5 that explained the majority of the phenotypic variation was in agreement with the QTL interval reported by Hernández Mora et al. (2017) using a PBA analysis in European peach germplasm. In the same chromosome, we also identified *qtnSSC_5.2* (0.7 Mb) and *qtnSSC_5.3* (13.0 Mb) whose positions matched QTLs mapped in previous studies using different germplasm and approaches (Nuñez-Lillo et al., 2019; Abdelghafar et al., 2020; Rawandoozi et al., 2020b). Furthermore, *qtnSSC_4.1* on chromosome 4 (6.6 Mb) was close to the QTL (*qSSC.V-Ch4-2007a*) detected by Zeballos et al. (2016), while *qtnSSC_4.3* was near *MD* locus reported by Eduardo et al. (2011). On the other hand, the *qtnSSC_1.1* (chromosome 1: 17.5 Mb) and *qtnSSC_6.1* (chromosome 6: 21.2 Mbp) were located in a different region in comparison to the QTLs or associated markers previously detected on those chromosomes (Fresnedo-Ramírez et al., 2015; Cao et al., 2016; Hernández Mora et al., 2017; Li et al., 2019; Shi et al., 2020). Concerning the traits pH and TA, the *qtnTA_5.1* and *qtnpH_5.1* collocated with the major locus for low-acid fruit (D-locus) previously reported in peach (Boudehri et al., 2009).

### Fruit Quality Hotspots in Peach Genome

Hotspot regions detected on chromosomes 1, 3, 4, 5, 6, and 8 controlled several fruit quality traits. The detection of hotspots in the genome indicates that genes related to certain traits are more densely concentrated in certain genomic regions (Zhang X. et al., 2019). The main hotspot on chromosome 4 (9.0–12.5 Mbp) included reliable QTNs for DAB, FW, RP, ADH, RD, FF, and SSC detected in different seasons and/or approaches and represents a target region for future breeding studies in peach. A QTL hotspot associated with quality traits was previously reported in peach on chromosome 4 (Cantín et al., 2010; Eduardo et al., 2011). However, the study was performed using SSR markers and the QTLs were detected in low-density linkage maps. Using high-density SNP maps, Rawandoozi et al. (2020a) reported QTLs for DAB and RD within the genetic region detected. Likewise, Hernández Mora et al. (2017) detected a hotspot for blush, SSC, RD and DAB in a wider genetic interval located at 11.2–14.1 Mbp in European germplasm. Moreover, Desnoues et al. (2016) identified a QTL hotspot in the same chromosome related to individual sugars and FW, although in a different location. In addition, the hotspot on chromosome 5 (0.3 to 3.7 Mbp) matched with the QTL hotspot for SSC and TA reported by Hernández Mora et al. (2017). Therefore, this study reinforces the importance of breeding programs targeting the improvement of fruit quality traits in peach focusing on the chromosome 4 and also demonstrated the necessity to promote further studies for the hotspot regions in chromosome 1, 3, 5, 6, and 8.

### Candidate Genes

Candidate genes (566) were identified within the haploblock regions encompassing the QTNs detected using the mrMLM 4.0 and FarmCPU, and the GO enrichment approach narrowed down the initial CG list (222) and revealed over-representation of certain GO terms (68). RNA binding proteins and serine-type endopeptidase inhibitor-related genes were identified, and previous studies revealed involvement in the regulation of flowering time (Steffen et al., 2019; Zhang F. et al., 2019). In addition, genes functionally annotated as 2-oxoglutarate-dependent dioxygenase, drug transmembrane transport, antiporter activity, pyridine nucleotide biosynthetic process, and chromatin assembly or disassembly were associated with fruit ripening in tomato, apricot, grape, peach, apple, and strawberry (Hanana et al., 2007; Farinati et al., 2017; Decros et al., 2019; Ding et al., 2020; García-Gómez et al., 2020). Furthermore, previous studies have shown that molybdopterin cofactor plays an important role in the metabolic control of avocado fruit growth and final fruit size (Cowan et al., 2001) and ion/H+ exchanger genes (GO: ion transmembrane transport) were critical for providing pH regulation (Pittman, 2012). Lastly, among the CG, *Prupe.4G262200* and *Prupe.4G261900* coding for endopolygalacturonases (GO: polygalacturonase activity) were previously involved in the inheritance of fruit texture and flesh adherence to the stone in peach (Peace et al., 2005; Gu et al., 2016).

Several CGs detected in our study have already been reported for productivity and fruit-related traits in peach. *Prupe.1G531600* (DAM5), *Prupe.1G531700* (DAM6), *Prupe.1G531500*, *Prupe.1G549600*, *Prupe.1G548000*, *Prupe.1G554100* were considered potential CG for bloom date in peach (Rawandoozi et al., 2020a). These genes are located within the hotspot region detected on chromosome 1 associated with BD and FF. Similarly, *Prupe.3G163100* (18.2 Mbp) located in the genetic interval of the hotspot on chromosome 3 was previously associated with blush and RP (redness around the stone) in peach (Frett et al., 2014; Zhang et al., 2018b; Guo et al., 2020). The main hotspot on chromosome 4 (9.0–12.5 Mbp) collocates with: *Prupe.4G186800*, the major locus controlling fruit ripening (Pirona et al., 2013) and CG for fruit flesh softening rate (Carrasco-Valenzuela et al., 2019); *Prupe.4G179900*, CG for RD and DAB (Elsadr et al., 2019); and *Prupe.4G185800* and *Prupe.4G187100* involved in anthocyanin biosynthesis and CGs for blush (Rawandoozi et al., 2020b). In addition, *Prupe_5G008400*, a CG controlling fruit acidity, is located within the hotspot (0.3–3.7 Mbp) on chromosome 5 (Wang et al., 2020).

## Conclusion

We successfully performed a multi-locus GWAS using mrMLM 4.0 and FarmCPU in 620 individuals from three public fresh market peach breeding programs. A total of 88 reliable QTNs were consistently detected in at least two seasons and/or in different methods. Hotspots for quality traits were identified on chromosomes 1, 3, 4, 5, 6, and 8. Candidate genes for quality traits were identified in the vicinity of the reliable QTNs detected using mrMLM 4.0 and FarmCPU. Furthermore, we observed that the position of the previously reported candidate genes for fruit-related traits (BD, Blush, DAB, ADH, RP, pH, and TA) matched with the position of the hotspots detected on chromosomes 1, 3, 4, and 5. Therefore, the information reported in this study supports the development of DNA tools for MAS in peach. Moreover, the importance of chromosome 4 hotspot in breeding for improvement of fruit quality is reinforced, and also emphasized the necessity to further study the hotspot regions on chromosomes 1, 3, 5, 6, and 8.

## Data Availability Statement

The datasets presented in this study can be found in online repositories. The names of the repository/repositories and accession number(s) can be found below: www.rosaceae.org, tfGDR1048/b.

## Author Contributions

CS: formal analysis and writing – original draft. LC: SNP data curation and review. WF: candidate genes analysis. MW, JC, and DB: resources and writing – review & editing. ZR: phenotypic analysis and review. KG: conceptualization, funding acquisition, resources, supervision, and writing – review & editing. All authors have read and approved the final manuscript.

## Conflict of Interest

The authors declare that the research was conducted in the absence of any commercial or financial relationships that could be construed as a potential conflict of interest.

## Footnotes

1
www.rosaceae.org
