# Corrigendum: Multi-Locus Genome-Wide Association Studies Reveal Fruit Quality Hotspots in Peach Genome

**DOI:** 10.3389/fpls.2022.879112

**Published:** 2022-03-17

**Authors:** Cassia da Silva Linge, Lichun Cai, Wanfang Fu, John Clark, Margaret Worthington, Zena Rawandoozi, David H. Byrne, Ksenija Gasic

**Affiliations:** ^1^Department of Plant and Environmental Sciences, Clemson University, Clemson, SC, United States; ^2^Department of Horticulture, Michigan State University, East Lansing, MI, United States; ^3^Department of Horticulture, University of Arkansas, Fayetteville, AR, United States; ^4^Department of Horticultural Sciences, Texas A&M University, College Station, TX, United States

**Keywords:** FarmCPU, mrMLM 4.0, candidate gene analyses, SNP array, RosBREED, QTN

In the original article, there was a mistake in **Supplementary Figure 2** as published. Within the **Supplementary Figure 2**, in the Legend the red line was labeled as “All.” The corrected label is “Admixed.”

In the original article, there was an error. In the manuscript, the term “broad sense heritability” should be replaced as “narrow sense heritability” in the three paragraphs shown below.

A correction has been made to **Materials and Methods**, “**Descriptive Analysis, Genetic Diversity, and Population Structure**,” 1:

“The descriptive analysis and the correlations between the traits were performed using the software Past (Hammer et al., 2001). The genetic diversity analysis was performed using the GenAlEx software (Peakall and Smouse, 2012). The narrow sense heritability was calculated using the R package Sommer (Covarrubias-Pazaran, 2016) using the h2.fun:”

A correction has been made to **Results**, “**Phenotypic Data**,” 3:

“The narrow sense heritability (h^2^) was estimated for all 14 traits (**Supplementary Table 4**). High average values of h^2^ (> 0.6) were observed for TA (0.87), RD (0.83), BD (0.77), ADH (0.77), FT (0.76), DAB (0.74), FDIA (0.73), SSC (0.72), Blush (0.71), FW (0.70), PW (0.70), RP (0.69), pH (0.69), and FF (0.68).”

A correction has been made to **Discussion**, 1:

“We have analyzed peach germplasm containing 620 individuals from three U.S. public fresh market breeding programs [University of Arkansas System Division of Agriculture (AR), Clemson University (SC) and Texas A&M University (TX)] for 14 traits over three seasons (2010, 2011, and 2012). Phenotypic variation was observed between individuals and seasons, and the mean values for BD, RD, FW, and SSC were lower than those reported in the Spanish and European germplasm (Hernández Mora et al., 2017; Font I Forcada et al., 2019). However, average values for RD and DAB observed in our study were in agreement with the values reported in the University of Guelph's peach germplasm, comprised of accessions originating from different regions across North America (Elsadr et al., 2019). A high and significant correlation between FW and FDIA (0.92) was previously observed in peach (da Silva Linge et al., 2015; Abdelghafar et al., 2020), as well as the positive correlation between RD and DAB (Elsadr et al., 2019) and the negative correlation between TA and pH (Abidi et al., 2011). In addition, the high estimated narrow sense heritability coefficients observed in this study ranging from 0.68 to 0.87, suggesting that the phenotypic variations of all traits are mainly affected by genetic factors, and therefore this dataset can be used for further genetic analyses.”

A correction has been made to **Results**, “**Genetic Variability, Population Structure, and Linkage Disequilibrium**,” 3:

“The LD decayed with increase of physical distance between SNPs in all groups (**Supplementary Figure 2**). Considering the admixed individuals, the average of *r*^2^ was 0.16. The physical distance over which LD decayed to half of its maximum value was around 540 kb. Different patterns of LD decays were observed in the three different groups. Group 3 revealed the highest average of *r*^2^ (0.32) and the longest physical distances in which LD decayed to half of its maximum value (1,620 kb), while group 2 showed shortest distance (480 kb). In the group 1, the LD decayed of its maximum value of *r*^2^ in ~540 kb.”

The authors apologize for this error and state that this does not change the scientific conclusions of the article in any way. The original article has been updated.

## Publisher's Note

All claims expressed in this article are solely those of the authors and do not necessarily represent those of their affiliated organizations, or those of the publisher, the editors and the reviewers. Any product that may be evaluated in this article, or claim that may be made by its manufacturer, is not guaranteed or endorsed by the publisher.
