# Supplementary material for: Multi-Locus Genome-Wide Association Studies Reveal Fruit Quality Hotspots in Peach Genome
Source: Front Plant Sci. 2021 Feb 25;12:644799. doi: 10.3389/fpls.2021.644799 (PMC7959719; doi:10.3389/fpls.2021.644799)
Supplement: Supplementary file 1 [file Image_1.pdf]

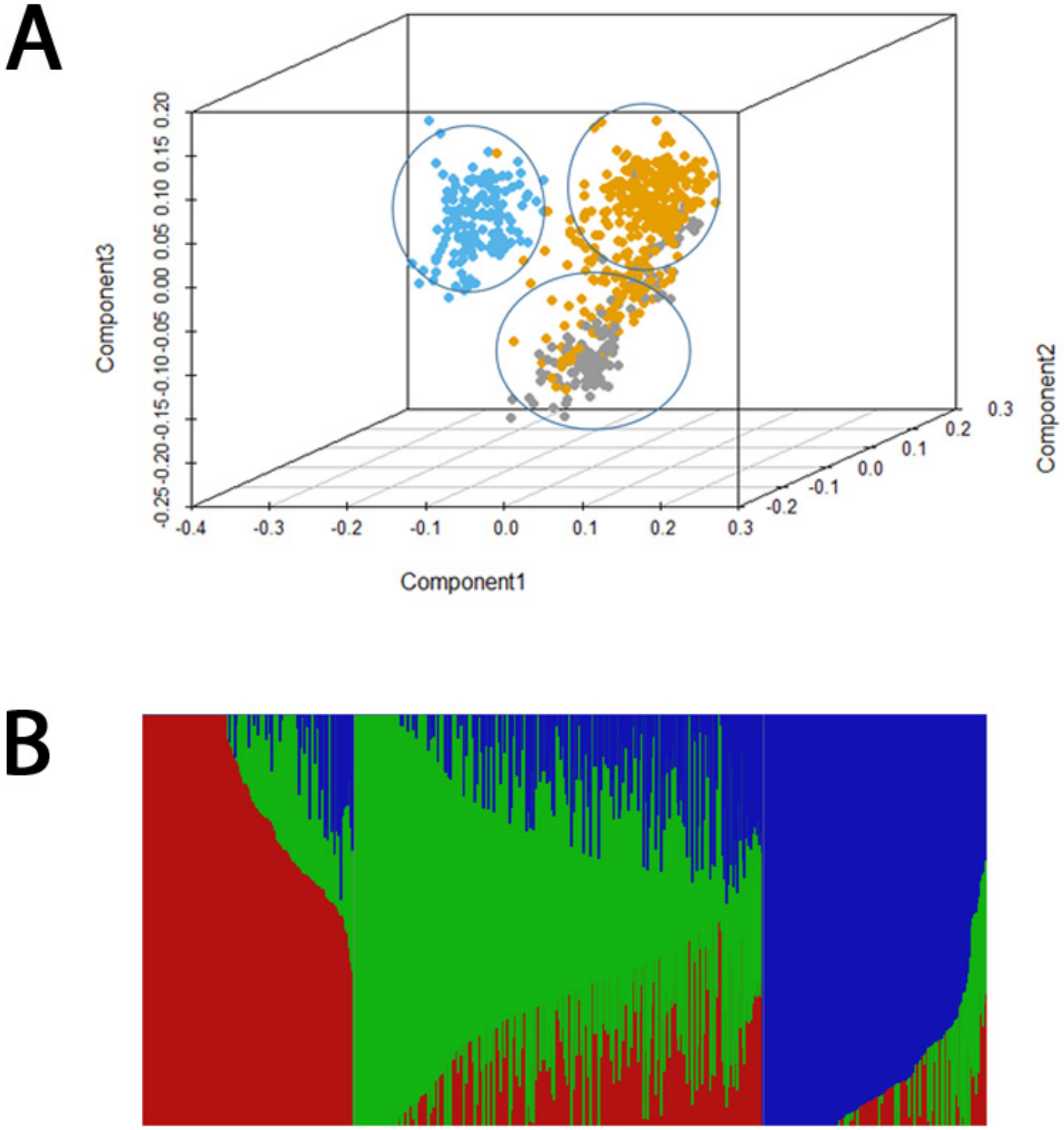

Supplementary Figure 1. Population structure observed in 620 individuals from three U.S. public fresh market peach breeding programs (Univ. of Arkansas, Texas A&M and Clemson Univ.) (A) Scatter plot of a Multidimensional Scaling analysis (MDS). The individuals were clustered into three groups, blue Texas A&M, orange Clemson Univ. and grey Univ. of Arkansas. (B) Population structure estimates (K = 3), the areas of the three colors (red, green and blue) illustrate the proportion of each subgroup. Group one - Texas A&M; group two - Clemson Univ.; and group three Univ. of Arkansas peach breeding program.
