# Supplementary figures and images for: Multi-Locus Genome-Wide Association Studies Reveal Fruit Quality Hotspots in Peach Genome
Source: Front Plant Sci. 2021 Feb 25;12:644799. doi: 10.3389/fpls.2021.644799 (PMC7959719; doi:10.3389/fpls.2021.644799)

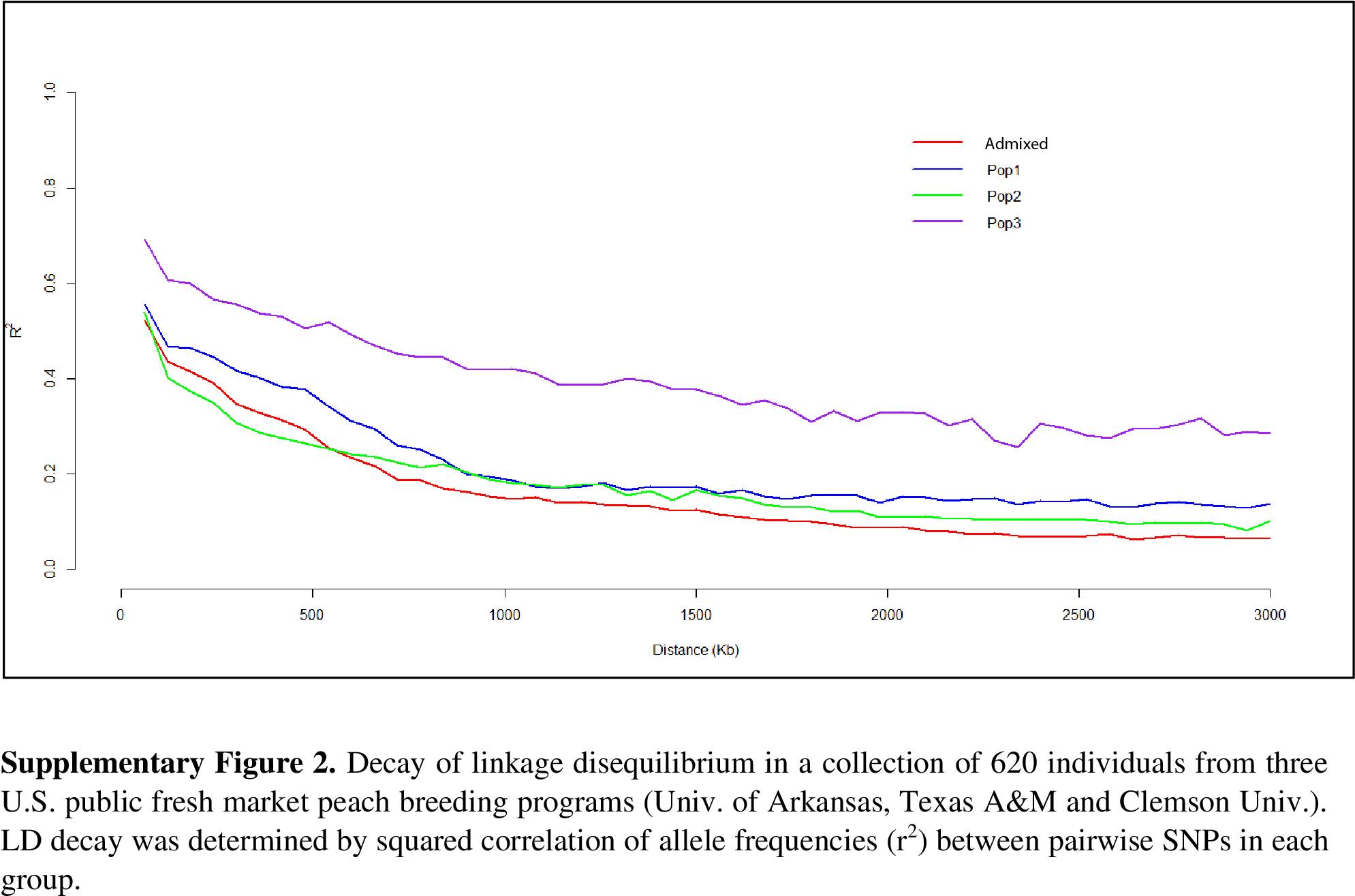

Supplement: Supplementary file 2 [file Image_2.tif]
